# Pigment Epithelium-Derived Factor Protects Retinal Neural Cells and Prevents Pathological Angiogenesis in an *Ex Vivo* Ischemia Model

**DOI:** 10.1155/2022/4199394

**Published:** 2022-08-05

**Authors:** Lei Xi, Marina Tikhonovich, Antje Biesemeier, Sylvie Julien-Schraermeyer, Ulrich Schraermeyer, Alexander V. Tschulakow

**Affiliations:** ^1^Division of Experimental Vitreoretinal Surgery, Centre for Ophthalmology, Institute for Ophthalmic Research, University of Tuebingen, Tuebingen, Germany; ^2^STZ Ocutox, Preclinical Drug Assessment, Hechingen, Germany

## Abstract

Ocular ischemia/hypoxia is a severe problem in ophthalmology that can cause vision impairment and blindness. However, little is known about the changes occurring in the existing fully formed choroidal blood vessels. We developed a new whole organ culture model for ischemia/hypoxia in rat eyes and investigate the effects of pigment epithelium derived factor (PEDF) protein on the eye tissues. The concentration of oxygen within the vitreous was measured in the enucleated rat eyes and living rats. Then, ischemia was mimicked by incubating the freshly enucleated eyes in medium at 4°C for 14 h. Eyes were fixed immediately after enucleation or were intravitreally injected with PEDF protein or with vehicle before incubation. After incubation, light and electron microscopy (EM) as well as Tunel staining was performed. In the living rats, the intravitreal oxygen concentration was on average at 16.4% of the oxygen concentration in the air and did not change throughout the experiment whereas it was ca. 28% at the beginning of the experiment and gradually decreased over time in the enucleated eyes. EM analysis revealed that the shape of the choriocapillaris changed dramatically after 14 h incubation in the enucleated eyes. The endothelial cells made filopodia-like projections into the vessel lumen. They appeared identical to the labyrinth capillaries found in surgically extracted choroidal neovascular membranes from patients with wet age-related macular degeneration (AMD). These filopodia-like projections nearly closed the vessel lumen and showed open gaps between neighboring endothelial cells. PEDF significantly inhibited labyrinth capillary formation and kept the capillary lumen open. The number of TUNEL-positive ganglion cells and inner nuclear layer cells was significantly reduced in the PEDF-treated eyes compared to the vehicle-treated eyes. The structural changes in the chroidal vessels observed under ischemia/hypoxia conditions can mimic early changes in the process of pathological angiogenesis as observed in wet AMD patients. This new model can be used to investigate short-term drug effects on the choriocapillaris after ischemia/hypoxia and it highlighted the potential of PEDF as a promising candidate for treating wet AMD.

## 1. Introduction

The eye is one of the highest energy-consuming organs [1] and thus has also one of the highest blood flows. The retinal vascular system supplies blood to the inner layers of the retina. The choroidal circulation has a particularly high blood flow, and a dense plexus of fenestrated capillaries supplies the outer third of the retina [2]. Because of the high blood flow, a reduction or disturbances in the structure of the choroidal vessels can lead to visual impairment and in severe cases result in blindness. Ocular ischemia is a typical symptom of diseases which affect the blood vessels of the eye such as occlusions, diabetic retinopathy (DR), or age-related macular degeneration (AMD) [3–5] and results in pathological changes of the vessels, VEGF overexpression, ocular neovascularization, growth of leaky vessels, and tissue damage. Previous studies on the effect of ischemia on choroidal vessels were mainly focused on choroidal perfusion and circulation [6], choroidal blood velocity, volume and flow [7], and effects on choroidal thickness and choroidal area [8]. However, there has not yet been a comprehensive study on the ultrastructural changes of choroidal vessels under ischemic conditions.

To investigate the pathogenesis of ocular ischemia, animal models have previously been developed by limiting the blood supply to the eye, e.g., by carotid artery (CCA) occlusion [9–11]. However, CCA severely interrupts blood supply to the brain and can lead to death [12]. On the other hand, individual differences in CCA are difficult to control. Thus, we established an ischemic *ex vivo* rat eye model by incubating freshly enucleated rat eyes in Dulbecco's modified eagle medium (DMEM) solution at 4°C for 14 hours. This provides controllable experimental conditions, in consideration of temperature and light. Additionally, the hypothermia reduces the metabolism and the oxygen consumption [13, 14] and slows down the physiological processes in the cells, and thus gives a higher temporal resolution for the analysis.

In order to study the changes of the oxygen concentration in the enucleated rat eyes, we measured the oxygen concentration in their vitreous close to the retina using an optical microsensor. The magnitude of the changes of the oxygen concentration in the preretinal vitreous was shown to correlate to that in the retina [15]. To be able to compare the oxygen concentration in our *ex vivo* model with that of the *in vivo* conditions, we additionally measured the oxygen concentration in the vitreous of living rats under isoflurane narcosis. In the living rats, the oxygen concentration was stable, while in the enucleated eyes, a slow but steady decrease of the oxygen concentration and consequently a hypoxia onset were detected.

This model was used to study the effects of PEDF protein on the hypoxia-induced changes in the eye tissues, especially in the choroidal vessels. PEDF is a promising treatment option for hypoxia-induced pathological changes in the vessels and CNV (choroidal neovascularization) especially because of its antiangiogenic and stabilizing functions [16–18]; additionally, it has important roles in other various physiological and pathophysiological processes like fibrogenesis, inflammation, neuroprotection, and stress response [19, 20]. Moreover, PEDF is a direct VEGF antagonist [21].

As mentioned, one of the consequences of ischemia is VEGF overexpression and thus neovascularization, which often leads to the growth of leaky vessels. Thus, anti-VEGF therapy is widely used to counteract this process. However, many side effects such as thrombotic microangiopathy in animal experiments and increased mortality in myocardial infarction (MI) patients have been reported [22, 23], and the therapy also interferes with the physiological effects of VEGF like balancing the survival, integrity, and maintenance of the homeostasis of choroidal endothelial cells [24, 25]. Additionally, there is a growing body of evidence linking anti-VEGF therapy with the development of geographic atrophy (GA), a symptom of advanced dry AMD [26–31]. PEDF being a VEGF antagonist in combination with its other mentioned features makes it a promising candidate for treating ischemia and CNV alone or in combination with other drugs.

## 2. Results

### 2.1. Intravitreal Oxygen Saturation in the Living Rats and the Incubated Eyes

**Figure 1:**
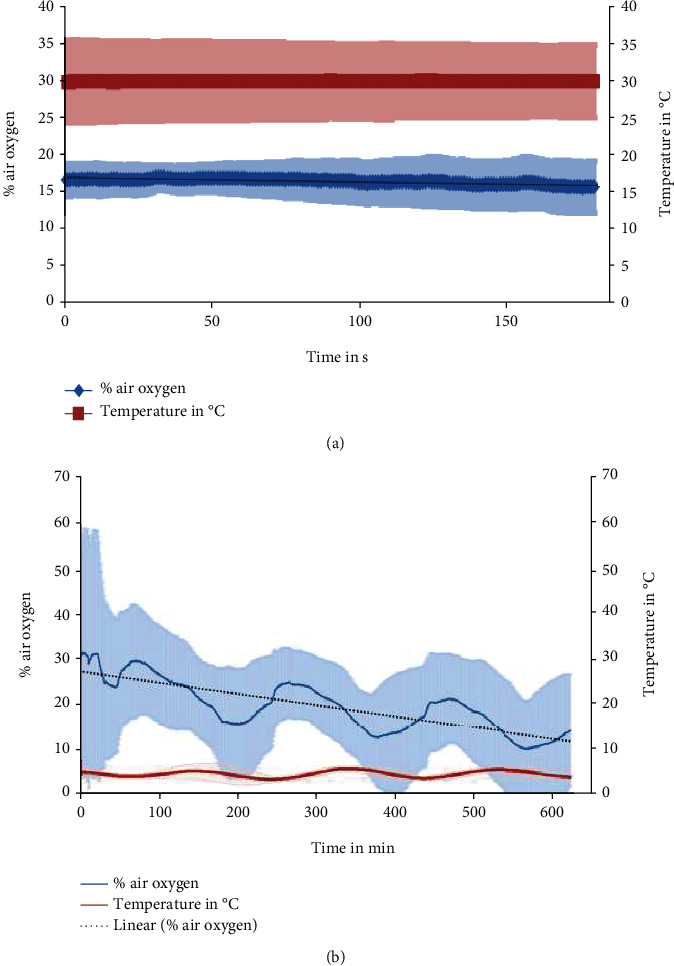
Results of oxygen concentration measurements. (a) *In vivo* (*n* =4) and (b) in the enucleated eyes (*n* =4). The results (mean and standard deviation) of the oxygen concentration measurements (blue) are shown in % of the oxygen in the air, and the temperature (orange) in °C. In (b), a linear trendline for the oxygen concentration measurements is shown as a black dotted line.

The value of oxygen concentration in the vitreous of living rats under isoflurane narcosis was on average stable at 16.4% of the oxygen concentration in the air. The body temperature was stable at 30° C (Figure 1(a)). The oxygen concentration in the vitreous of the incubated eyes was continuously sinking and fell to half of the initial value which was ca. 28% within approximately 600 minutes (Figure 1(b)).

### 2.2. Microstructural Analysis

**Figure 2:**
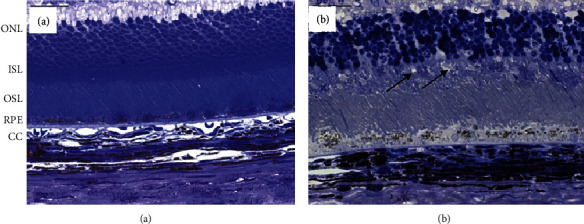
Representative light micrographs of control rat retinae of a fresh enucleated and immediately fixed eye (a) and an eye which was incubated for 14 h at 4°C (b). Compared to the control retina, the photoreceptor inner segment layer (IS) was slightly swollen and disorganized, as was the retinal pigment epithelium (RPE). ONL: outer nuclear layer; ISL: inner photoreceptor segments layer; OSL: outer photoreceptor segments layer; RPE: retinal pigmented epithelium; CC: choriocapillaris. Bar =50 *μ*m.

Compared to the control group (Figure 2(a)), the photoreceptors and RPE cells of the incubated eyes were slightly swollen and disorganized (Figure 2(b)). Detailed information of changes in the retinal and choroidal vasculature was not observed at this level of magnification.

### 2.3. Cell Death Assay

**Figure 3:**
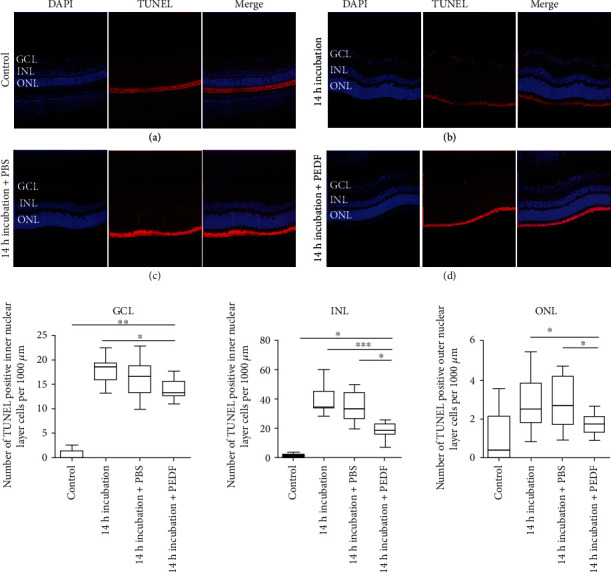
(a–d) Representative images of the TUNEL experiments for eyes which were fixed immediately after enucleation (control, *n* = 5) (a), 14 h of incubation at 4°C (b), 14 h of incubation after PBS treatment (*n* = 5) (c), and 14 h of incubation 4°C after PEDF treatment (*n* = 5) (d) (red - TUNEL-positive cells, blue - DAPI stained cell nuclei, merge - overlay of the TUNEL and DAPI images). Lower part: statistical analysis for the TUNEL-positive cells in the ganglion cell layer (GCL), inner nuclear layer (INL), and outer nuclear layer (ONL) per 1000 *μ*m retina length. The data represent the mean ± SD (standard deviation). All treatment groups were compared to the PEDF-treated group using ANOVA with a Kruskal-Wallis post-test (∗*p* < 0.05, ∗∗*p* < 0.001, ∗∗∗*p* < 0.0001). Scale bar =50 *μ*m.

In the eyes which were fixed immediately after enucleation (Figure 3(a)), hardly any TUNEL-positive cells were found. Compared to that, the number of TUNEL-positive cells in all layers (GCL, INL, and ONL) was significantly increased in the 14-hour untreated and the vehicle-treated groups (Figures 3(b) and 3(c)). In the PEDF-treated group, the number of TUNEL-positive cells per 1000 *μ*m was significantly reduced in all layers (GCL, INL, and ONL) compared to the 14-hour untreated and the vehicle-treated groups (Figure 3(d)).

### 2.4. Ultrastuctural Analysis

**Figure 4:**
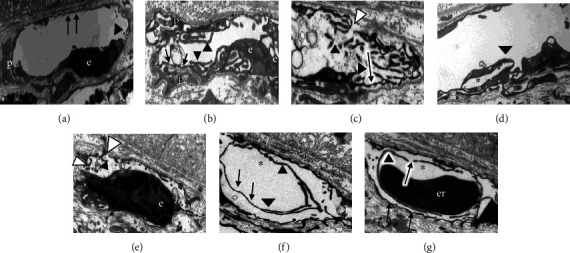
Electron micrographs of choriocapillaris vessels. (a) From a rat eye which was fixed immediately after enucleation: regular endothelium with fenestrations (marked by black arrows). There are a few unsuspicious small projections to the vessel lumen which are indicated by a black arrowhead (same labels used also in the following images). (b–g) From eyes after 14 h of incubation at 4°C with hypoxia-induced changes: filopodia-like projections from the endothelial cells (e) into the vessel lumen (black arrowhead) in (b–g), which are very prominent in (b) and (c) and form labyrinth-like structures; in (f) and (g), the projections are particularly long; in (d), (f), and (g), they form new lumen in the vessel (marked with ∗); pericytes (p) which also have projections to the surrounding tissue; endothelial gaps (marked with white arrowheads); fenestrations (marked by black arrows). er: erythrocyte, scale bar = 2 *μ*m.

The most prominent ultrastructural changes in the eyes after 14 hours of incubation at 4°C compared to the eyes fixed immediately after enucleation (Figure 4(a)) were observed in the choriocapillaris vessels. The following changes were observed: Choriocapillaris (CC) vessels contained fenestrations not only on the wall facing the RPE but also very prominently on the opposite side (Figures 4(c) and 4(g)), endothelial cells contained filopodia-like projections in the vessel lumen (Figures 4(b)–4(e) and 4(g)), the filopodia-like projections formed a labyrinth-like structure which nearly closed the vessel lumen (Figures 4(b)–4(d)), the filopodia-like projections often had fenestrations (Figures 4(f) and 4(g) and 4(h)), the filopodia-like projections were often associated with erythrocytes or completely surrounded them (Figure 4(g)), the vessel lumens had openings towards the interstitium (Figures 4(c) and 4(e)), pericytes also became elongated and produced filopodia-like projections which pointed to the surrounding tissue (Figure 4(b)), there were vessels with endothelial cells or filopodias which attempted to build up a new lumen inside the existing one (Figures 4(d) and 4(f)), and Bruch's membrane contained cells (Figures 4(d) and 4(f)).

**Figure 5:**
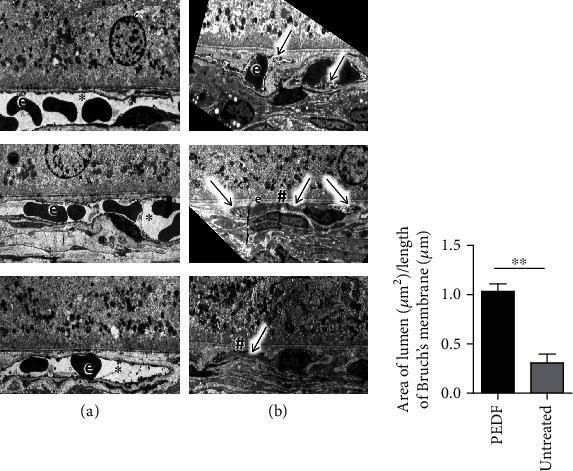
Electron micrographs of representative choriocapillaris vessels. (a) From rat eyes which were intravitreally injected with PEDF after enucleation and was incubated for 14 h at 4°C: only small projections and normal vessel lumen (∗). (b) From an eyes which were incubated for 14 h at 4°C: here the lumen of the capillaries were, reduced, due to “labyrinth” formation (arrows) or even collapsed (#). e: erythrocyte, scale bar = 5 *μ*m. Quantification: analysis of the ratios of the area of the vessels lumina divided by the length of the Bruch's membrane for the PEDF and untreated eyes (*n* = 5 for each group, the mean and standard deviation are shown). For statistical analysis a Students *t*-test was used (∗∗*p* < 0.001).

In the untreated eyes, the vessel lumen was reduced or completely collapsed (Figure 5(b)). In the PEDF-treated eyes, the choroidal vessel lumen was wider with no or little labyrinth capillaries (Figure 5(a)).

### 2.5. Analysis of the Ratios of the Area of the Vessel Lumen Divided by the Length of Bruch's Membrane for the PEDF- and untreated Eyes

There was a significant difference (*P* < 0.001) between the ratios of the area of the vessel lumina of the PEDF- (1.034 ± 0.077 *μ*m^2^/*μ*m) and untreated (0.308 ± 0.087 *μ*m^2^/*μ*m) groups (Figure 5 lower part).

## 3. Discussion

The main currently used methods for clinical treatment of retinal ischemia are laser photocoagulation and intravitreal administration of antiangiogenic agents targeting VEGF. However, both treatment options are unable to heal the condition and only limit the consequential damage induced by VEGF overexpression and CNV formation [32] and have severe side effects. Laser photocoagulation of the ischemic retina reduces the expression of VEGF caused by ischemia, but the thermal effect of the laser can damage the adjacent retina [33]. It has been shown that the use of anti-VEGF therapy can induce side effects like thrombosis and infarct and consequently increase the mortality [22, 34, 35]. Moreover, the long-term treatment can cause loss of choriocapillaris and geographic atropy [36]. Thus, new therapy approaches for the treatment of retinal ischemia are urgently needed and suitable models are required for the development of such approaches.

The models which simulate the situation in the patient in the closest way are *in vivo* animal models. Several models have been developed for the study of ocular ischemia, e.g., laser occlusion of the retinal veins [37], permanent unilateral common carotid artery occlusion (UCCAO) [10], transient bilateral common carotid artery occlusion (tBCCAO) [38], and elevation of intraocular pressure to cut off the blood supply from the retinal artery [39]. The disadvantage of these models is that they have a high intraindividual variation. Additionally, the current social ethics advocates a reduction of the use of laboratory animals.

*In vitro* studies of ocular ischemia usually use cell cultures or organotypic retinal cultures and chemical solutions like N_2_-saturated PBS containing iodoacetic acid [40], PBS containing iodoacetic acid and sodium cyanide [41], or cobalt chloride (CoCl_2_) [42] to induce hypoxia in the cultures. Cell culture based experiments limit the analysis of the mechanisms to only one or a few cell types. Even organotypic retinal culture explants can only partly simulate the processes, because the integrity of the eye is destroyed during the preparation. Additionally, in these *in vitro* approaches, a chemical induction of hypoxia is used, which cannot completely simulate the real ischemic/hypoxic conditions during the process of retinal diseases as it has additional toxic effects on the cultured cells [43–45].

Our established *ex vivo* whole eye ischemia model has the advantage that it is easy to reproduce and the experimental conditions such as temperature and light are well controllable. Furthermore, the usage of the whole eyeball avoids preparation artifacts and keeps the integrity of the eye and its microenvironment.

To study the oxygen conditions in the enucleated eyes, we started with a reference measurement in living rats. Our oxygen concentration measurements *in vivo* were performed in rats under inhalation narcosis with 3.5% isoflurane. The rats' oxygen concentration in the vitreous was stable at 16.4% of the air oxygen concentration, and the body temperature was stable at 30°C. The normal body temperature of a rat is considered to be ca. 37.5°C which is 25% more than under narcosis. How exactly the narcosis influences the oxygen concentration in the vitreous remains unclear.

In the freshly enucleated eyes, the mean oxygen concentration in the vitreous was ca. 28% of the air oxygen concentration at the earliest time point of measurement, which is 65% more than that in the *in vivo* rat eyes under isoflurane narcosis. The oxygen concentration in the vitreous was slowly but steadily sinking and fell to half of the value at the earliest time point of measurement, directly after enucleation within approximately 600 minutes. The temperature in the refrigerator was at average 4°C fluctuating in a sinusoidal way between ca. 3 and 5°C within a period of ca. 200 min. The oxygen concentration in the vitreous also fluctuated within the same period but with a 50 min. time shift, so that after a local temperature minimum, a local oxygen concentration maximum followed. This fluctuation turned out to be a measuring artifact because the same fluctuation was present if the measurement took place in medium or water in the refrigerator (data not shown).

The drop of the oxygen concentration in the enucleated eyes induced hypoxia in the eye tissues, without the administration of any chemical agents, evidenced by the occurrence of typical hypoxia-induced pathological changes in the choroid and the death of ganglion cells.

The neuroprotective effect of PEDF on the retinal neurons has been confirmed in previous studies [46, 47] as was shown in our study. PEDF treatment significantly reduced the number of TUNEL-positive ganglion cells and inner and outer nuclear cells after 14 h of incubation compared to no treatment or PBS treatment (Figure 3). Retinal neurons, especially the ganglion cells, are very sensitive to hypoxia. Ganglion cells are known to start dying after one hour of hypoxic conditions [48]. The neuroprotective effect of PEDF might be a promising approach which could help to prevent ischemic retinopathy.

The most striking changes during our EM analysis in the rat eyes after 14 hours incubation at 4°C were found in the choroidal vessels, especially filopodia-like endothelial projections, which occurred in their lumen (Figure 4). These projections were similar to those which we found in “labyrinth capillaries” described by us previously in human CNV membranes [49]. These pathological capillaries are probably responsible for the permanent leakage and edema formation, as the leaky sites in this kind of labyrinth capillary cannot be closed by thrombocytes because these are unable to move to the leaky site due to the reduced lumen [49]. Thus, our model can be used for the study of the formation mechanisms of labyrinth capillaries and to test approaches to suppress their formation. One approach was successfully tested in this study. We showed that a single intravitreal PEDF injection inhibits the formation of filopodia-like projections. Moreover, PEDF treatment preserved the vessel structure and prevented the collapse of the vessel lumen which was observed in untreated eyes (Figure 5). The exact mechanism of this process is not clear, but there is some evidence that PEDF has a protective effect on podocyte structural integrity and preserves the organization of the actin cytoskeleton [50]. Moreover, PEDF being a VEGF antagonist inhibits neovascularization and prevents VEGF-induced changes in endothelial cells [51, 52].

In conclusion, by using the described *ex vivo* whole eye organ culture method, ultrastructural changes such as filopodia-like projections in the choroid can be induced, resembling ischemia-induced morphological changes in vessels similar to that in early CNV. PEDF can significantly improve choroidal vascular status, inhibit the formation of the filopodia-like projections under ischemic conditions, and maintain the morphology of choroidal blood vessels. Furthermore, PEDF protects retinal neuronal cells from apoptosis under ischemic conditions.

## 4. Materials and Methods

### 4.1. Animals and Ethics Statement

For the study, adult Long Evans rats (Janvier Labs, France) were used. The animals were kept in air-conditioned rooms on a 12 h light-dark cycle and had free access to water and food. The experiments were conducted in accordance with the ARVO statement for use of animals in ophthalmic and visual research and were in compliance with §4 section 3 of the German law on animal protection. They were reviewed and approved by the “Einrichtung für Tierschutz, Tierärztlichen Dienst und Labortierkunde, Tübingen.” The rats were sacrificed by exposure to isoflurane in their cages until their breathing stopped, and then left for an additional minute, after that the animals were euthanized by cervical dislocation.

### 4.2. Investigation of the Oxygen Pressure in the Vitreous of the Living Rats

**Figure 6:**
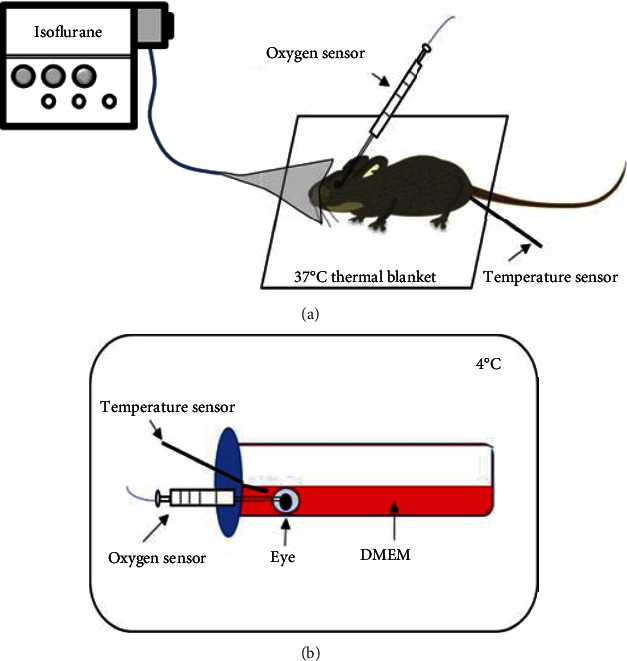
Schematic setup of the measurement of the oxygen concentration in the vitreous of the living rats and in the enucleated rat eyes. Measurement method *in vivo* (a) and *ex vivo* (b).

The rats were anesthetized using an isoflurane narcosis unit. The unit provided 3.5% isoflurane-air mixture into a mask fixed on the rat`s head. The rat was kept on a cushion at 37°C. For the measurements, we used the WPI oxygen micro unit (WPI, Sarasota USA) according to the manufacturer's instructions. After application of local anesthesia on the eye (Novesine, Puchheim, Germany) and puncturing of the sclera with a sharp needle, the syringe-like oxygen sensor (BioOxy Sensor, WPI) fixed on a micromanipulator system was inserted into the vitreous of the rats eye. The system's own temperature sensor (°C) was applied rectally (Figure 6(a)). After the experiment, the animals were euthanized and the unanalyzed eye was used for the *ex vivo* analysis. Four rats were used for the analyses.

### 4.3. Investigation of the Oxygen Pressure in the Vitreous of the Enucleated Eyes

The same WPI oxygen micro unit was used for the *ex vivo* studies. After the enucleation of the rat'`s eye, a hole was punctured immediately in the eye behind the corneal limbus using a sharp needle, and the oxygen microsensor was inserted into the vitreous. Then, the eye was put in a 50 mL tube filled with 25 mL of DMEM solution and kept at 4°C in the refrigerator for 14 hours. The temperature sensor was kept in the medium (Figure 6(b)). Four eyes were used for this project part.

### 4.4. *Ex Vivo* Whole Eye Model

To establish the model, seven rats were used. Immediately after the rats were sacrificed, they were enucleated and the eyes were immersed for 30 seconds in 70% ethanol in order to eliminate microorganisms on the outer surface. Seven eyes were put in DMEM and kept at 4°C for 14 hours. The other seven eyes were enucleated and fixed immediately in glutaraldehyde or in formalin for electron microscopical (EM) and light microscopical analyses, respectively, and used as controls.

### 4.5. Intravitreal Injection

Intravitreal injections into the enucleated eyes were performed using a 10 *μ*l NanoFil syringe (WPI). A volume of 5 *μ*l PBS alone or 5 *μ*l PBS with 10 *μ*g of PEDF protein was injected into the vitreous of each eye through a puncture on the sclera 1 mm from the limbus, directly before incubation. For each treatment group, five animals were used.

### 4.6. Sample Preparation for TUNEL Analysis and Immunohistochemistry

The enucleated eyes were fixed in 4.5% formaldehyde (Carl Roth, Karlsruhe, Germany) immediately or after incubation, embedded in paraffin, cut in 4 *μ*m-thick sections, and mounted on slides according to standard protocols.

### 4.7. Terminal Deoxynucleotidyl Transferase- (TdT-) Mediated dUTP Nick End Labeling (TUNEL) Analysis

Cell death was assessed using the TUNEL assay. A commercial TUNEL kit (*in situ* cell death detection with fluorescein; Roche Biochemicals, Mannheim, Germany) was used according to the manufacturer's instructions. The stained slides were photographed with an Imager Z2 ApoTome microscope (Carl Zeiss Microscopy GmbH, Oberkochen, Germany). The numbers of TUNEL-positive cells in the ganglion cell layer (GCL), in the inner and outer nuclear layers (INL and ONL, respectively) per 1000 *μ*m retina were calculated for each group.

### 4.8. Sample Preparation for Light and Electron Microscopy

For light and electron microscopy, eyes were fixed at 4°C in 5% glutaraldehyde in 0.1 M cacodylate buffer (pH 7.4). After washing in cacodylate buffer, the cornea and lens were removed and eye cups were hemisected. The halves were post-fixed in 1% osmium tetroxide in 0.1 M cacodylate buffer and bloc-stained with uranyl acetate. The samples were dehydrated and embedded in Epon following standard procedures using reagents from AppliChem (Darmstadt, Germany), Merck (Darmstadt, Germany), and Serva (Heidelberg, Germany). Light microscopy on toluidine blue stained semi-thin sections (500 nm) was performed with a Zeiss Axioskop (Zeiss, Jena, Germany). Ultrathin sections (70 nm) were mounted on copper slot grids (Plano, Wetzlar, Germany) and stained with lead citrate and examined with a Zeiss 900 electron microscope (Zeiss, Jena, Germany).

### 4.9. Analysis of the Ratios of the Area of the Vessel Lumina and Divided by the Length of Bruch's Membrane for the PEDF and untreated Eyes

Electron micrographs (20000 X magnification) from the choriocapillaris in ten randomly selected areas in proximity to the optic nerve from each of the three PEDF- and untreated eyes were analyzed. The total area of the lumen were calculated. The results of the area were divided by the corresponding length of Bruch's membrane associated with the vessels, to gain the area/*μ*m of Bruch's membrane value and to make these values comparable.

### 4.10. Statistical Analysis

The statistical analyses were performed using the GraphPad Prism 5 software. The results of the area of vessel lumen and the area of endothelial cells measurements were analyzed using Student's *t* test. The results of TUNEL experiments were analyzed using ANOVA with a Kruskal-Wallis post-test.

## Data Availability

The raw data are available from the corresponding author upon request.

## Additional Points

*Summary Statement*. PEDF protects retinal neural cells and prevents the formation of pathological choroidal neovascularization-like choriocapillaris in an *ex vivo* ischemia model: implication for the treatment of wet AMD.

## References

[1] Yu D. Y., Cringle S. J. (2001). Oxygen distribution and consumption within the retina in vascularised and avascular retinas and in animal models of retinal disease. *Progress in Retinal and Eye Research*.

[2] Campochiaro P. A. (2015). Molecular pathogenesis of retinal and choroidal vascular diseases. *Progress in Retinal and Eye Research*.

[3] Coleman D. J., Silverman R. H., Rondeau M. J., Lloyd H. O., Khanifar A. A., Chan R. V. P. (2013). Age-related macular degeneration: choroidal ischaemia. *British Journal of Ophthalmology*.

[4] Dong A., Xie B., Shen J. (2009). Oxidative stress promotes ocular neovascularization. *Journal of Cellular Physiology*.

[5] Roy S., Kern T. S., Song B., Stuebe C. (2017). Mechanistic insights into pathological changes in the diabetic retina: implications for targeting diabetic retinopathy. *American Journal of Pathology*.

[6] Wakabayashi T., Ikuno Y. (2010). Choroidal filling delay in choroidal neovascularisation due to pathological myopia. *British Journal of Ophthalmology*.

[7] Grunwald J. E., Metelitsina T. I., DuPont J. C., Ying G. S., Maguire M. G. (2005). Reduced foveolar choroidal blood flow in eyes with increasing AMD severity. *Investigative Ophthalmology & Visual Science*.

[8] Kang H. M., Choi J. H., Koh H. J., Lee S. C. (2019). Significant changes of the choroid in patients with ocular ischemic syndrome and symptomatic carotid artery stenosis. *PLoS One*.

[9] Lavinsky D., Arterni N. S., Achaval M., Netto C. A. (2006). Chronic bilateral common carotid artery occlusion: a model for ocular ischemic syndrome in the rat. *Graefes Archive for Clinical and Experimental Ophthalmology*.

[10] Lee D., Kang H., Yoon K. Y., Chang Y. Y., Song H. B. (2020). A mouse model of retinal hypoperfusion injury induced by unilateral common carotid artery occlusion. *Experimental Eye Research*.

[11] Yamamoto H., Schmidt-Kastner R., Hamasaki D. I., Yamamoto H., Parel J. M. (2006). Complex neurodegeneration in retina following moderate ischemia induced by bilateral common carotid artery occlusion in Wistar rats. *Experimental Eye Research*.

[12] Crespo-Garcia S., Reichhart N., Skosyrski S. (2018). Individual and temporal variability of the retina after chronic bilateral common carotid artery occlusion (BCCAO). *PLoS One*.

[13] Coassin M., Duncan K. G., Bailey K. R., Singh A., Schwartz D. M. (2010). Hypothermia reduces secretion of vascular endothelial growth factor by cultured retinal pigment epithelial cells. *British Journal of Ophthalmology*.

[14] Takeyama M., Yoneda M., Gosho M., Iwaki M., Zako M. (2015). Decreased VEGF-A and sustained PEDF expression in a human retinal pigment epithelium cell line cultured under hypothermia. *Biological Research*.

[15] Cringle S. J., Yu D. Y. (2004). Intraretinal oxygenation and oxygen consumption in the rabbit during systemic hyperoxia. *Investigative Ophthalmology & Visual Science*.

[16] Gehlbach P., Demetriades A. M., Yamamoto S. (2003). Periocular injection of an adenoviral vector encoding pigment epithelium- derived factor inhibits choroidal neovascularization. *Gene Therapy*.

[17] Mori K., Duh E., Gehlbach P. (2001). Pigment epithelium-derived factor inhibits retinal and choroidal neovascularization. *Journal of Cellular Physiology*.

[18] Saishin Y., Silva R. L., Saishin Y. (2005). Periocular gene transfer of pigment epithelium-derived factor inhibits choroidal neovascularization in a human-sized eye. *Human Gene Therapy*.

[19] Brook N., Brook E., Dharmarajan A., Chan A., Dass C. R. (2019). The role of pigment epithelium-derived factor in protecting against cellular stress. *Free Radical Research*.

[20] He X. M., Cheng R., Benyajati S., Ma J. X. (2015). PEDF and its roles in physiological and pathological conditions: implication in diabetic and hypoxia-induced angiogenic diseases. *Clinical Science*.

[21] Zhang S. X., Wang J. J., Gao G., Parke K., Ma J. X. (2006). Pigment epithelium-derived factor downregulates vascular endothelial growth factor (VEGF) expression and inhibits VEGF-VEGF receptor 2 binding in diabetic retinopathy. *Journal of Molecular Endocrinology*.

[22] Hanhart J., Comaneshter D. S., Freier-Dror Y., Vinker S. (2018). Mortality associated with bevacizumab intravitreal injections in age-related macular degeneration patients after acute myocardial infarct: a retrospective population-based survival analysis. *Graefes Archive for Clinical and Experimental Ophthalmology*.

[23] Schraermeyer U., Julien S. (2012). Formation of immune complexes and thrombotic microangiopathy after intravitreal injection of bevacizumab in the primate eye. *Graefes Archive for Clinical and Experimental Ophthalmology*.

[24] Ibuki M., Lee D., Shinojima A., Miwa Y., Tsubota K., Kurihara T. (2020). Rice bran and vitamin B6 suppress pathological neovascularization in a murine model of age-related macular degeneration as novel HIF inhibitors. *International Journal of Molecular Sciences*.

[25] Semeraro F., Morescalchi F., Duse S., Gambicorti E., Cancarini A., Costagliola C. (2015). Pharmacokinetic and pharmacodynamic properties of anti-VEGF drugs after intravitreal injection. *Current Drug Metabolism*.

[26] Cho H. J., Lee T. G., Han S. Y. (2016). Long-term visual outcome and prognostic factors of intravitreal anti-vascular endothelial growth factor treatment for retinal angiomatous proliferation. *Graefe’s Archive for Clinical and Experimental Ophthalmology*.

[27] Fleckenstein M., Keenan T. D. L., Guymer R. H. (2021). Age-related macular degeneration. *Nature Reviews. Disease Primers*.

[28] Gemenetzi M., Lotery A. J., Patel P. J. (2017). Risk of geographic atrophy in age-related macular degeneration patients treated with intravitreal anti-VEGF agents. *Eye (London, England)*.

[29] Kaynak S., Kaya M., Kaya D. (2018). Is there a relationship between use of anti-vascular endothelial growth factor agents and atrophic changes in age-related macular degeneration patients. *Turkish Journal of Ophthalmology*.

[30] Plyukhova A. A., Budzinskaya M. V. (2018). The role of anti-VEGF therapy in geographic atrophy progression. *Vestnik Oftalmologii*.

[31] Spooner K. L., Fraser-Bell S., Cozzi M. (2020). Macular atrophy incidence and progression in eyes with neovascular age-related macular degeneration treated with vascular endothelial growth factor inhibitors using a treat-and-extend or a pro re nata regimen: four-year results of the MANEX study. *Ophthalmology*.

[32] Jalilian E., Elkin K., Shin S. R. (2020). Novel cell-based and tissue engineering approaches for induction of angiogenesis as an alternative therapy for diabetic retinopathy. *International Journal of Molecular Sciences*.

[33] Shimada Y., Shibuya M., Shinoda K. (2021). Transient increase and delay of multifocal electroretinograms following laser photocoagulations for diabetic macular edema. *Clinical Medicine*.

[34] Hanhart J., Comaneshter D. S., Freier Dror Y., Vinker S. (2017). Mortality in patients treated with intravitreal bevacizumab for age-related macular degeneration. *BMC Ophthalmology*.

[35] Hanhart J., Comaneshter D. S., Vinker S. (2018). Mortality after a cerebrovascular event in age-related macular degeneration patients treated with bevacizumab ocular injections. *Acta Ophthalmologica*.

[36] Schutze C., Wedl M., Baumann B., Pircher M., Hitzenberger C. K., Schmidt-Erfurth U. (2015). Progression of retinal pigment epithelial atrophy in antiangiogenic therapy of neovascular age-related macular degeneration. *American Journal of Ophthalmology*.

[37] Neo T., Gozawa M., Takamura Y., Inatani M., Oki M. (2020). Gene expression profile analysis of the rabbit retinal vein occlusion model. *PLoS One*.

[38] Lee D., Miwa Y., Jeong H. (2020). A murine model of ischemic retinal injury induced by transient bilateral common carotid artery occlusion. *Jove-Journal of Visualized Experiments*.

[39] Lin C. M., Titchenell P. M., Keil J. M. (2018). Inhibition of atypical protein kinase C reduces inflammation-induced retinal vascular permeability. *American Journal of Pathology*.

[40] Catalani E., Cervia D., Martini D. (2007). Changes in neuronal response to ischemia in retinas with genetic alterations of somatostatin receptor expression. *European Journal of Neuroscience*.

[41] Mastrodimou N., Lambrou G. N., Thermos K. (2005). Effect of somatostatin analogues on chemically induced ischaemia in the rat retina. *Naunyn-Schmiedebergs Archives of Pharmacology*.

[42] Tsai T., Mueller-Buehl A. M., Satgunarajah Y., Kuehn S., Dick H. B., Joachim S. C. (2020). Protective effect of the extremolytes ectoine and hydroxyectoine in a porcine organ culture. *Graefes Archive for Clinical and Experimental Ophthalmology*.

[43] Hurst J., Mueller-Buehl A. M., Hofmann L. (2020). iNOS-inhibitor driven neuroprotection in a porcine retina organ culture model. *Journal of Cellular and Molecular Medicine*.

[44] Karovic O., Tonazzini I., Rebola N. (2007). Toxic effects of cobalt in primary cultures of mouse astrocytes: similarities with hypoxia and role of HIF-1*α*. *Biochemical Pharmacology*.

[45] Tang M. J., Yang Y., Yu J. Z. (2017). Discordant mRNA and protein expression of CXCR4 under in vitro CoCl2-induced hypoxic conditions. *Biochemical and Biophysical Research Communications*.

[46] Burger S., Meng J., Zwanzig A. (2021). Pigment epithelium-derived factor (PEDF) receptors are involved in survival of retinal neurons. *International Journal of Molecular Sciences*.

[47] Valiente-Soriano F. J., Di Pierdomenico J., Garcia-Ayuso D. (2020). Pigment epithelium-derived factor (PEDF) fragments prevent mouse cone photoreceptor cell loss induced by focal Phototoxicity in vivo. *International Journal of Molecular Sciences*.

[48] Selles Navarro I., Villegas Perez M. P., Salvador Silva M., Ruiz Gomez J. M., Vidal Sanz M. (1996). Retinal ganglion cell death after different transient periods of pressure-induced ischemia and survival intervals - a quantitative in vivo study. *Investigative Ophthalmology & Visual Science*.

[49] Schraermeyer U., Julien S., Biesemeier A., Bartz-Schmidt K. U., Wolburg H. (2015). A new kind of labyrinth-like capillary is responsible for leakage from human choroidal neovascular endothelium, as investigated by high-resolution electron microscopy. *Graefe's Archive for Clinical and Experimental Ophthalmology*.

[50] Awad A. S., Gao T., Gvritishvili A. (2013). Protective role of small pigment epithelium-derived factor (PEDF) peptide in diabetic renal injury. *American Journal of Physiology-Renal Physiology*.

[51] Ogata N., Wang L., Jo N. (2001). Pigment epithelium derived factor as a neuroprotective agent against ischemic retinal injury. *Current Eye Research*.

[52] Stellmach V., Crawford S. E., Zhou W., Bouck N. (2001). Prevention of ischemia-induced retinopathy by the natural ocular antiangiogenic agent pigment epithelium-derived factor. *Proceedings of the National Academy of Sciences of the United States of America*.

